# Supplementary material for: Are carbon emissions trading and green financial instruments synergistic? -Comprehensive quantitative research based on content analysis
Source: PLoS One. 2024 Mar 7;19(3):e0298601. doi: 10.1371/journal.pone.0298601 (PMC10919601; doi:10.1371/journal.pone.0298601)
Supplement: S1 Data — (ZIP) [file pone.0298601.s001.zip › Data/地级市Z_F工作报告.zip!/║■─╧/╘└╤⌠╩╨/└┤╫╘╣1⁄2╓┌║┼í╛┬φ┐╦╩2╛▌═°í┐_2013.docx]

各位代表：

　　现在，我代表市人民政府，向大会报告政府工作，请予审议，并请市政协委员和其他列席人员提出意见。

　　过去五年工作回顾

　　五年来，在中共岳阳市委的坚强领导下，我们坚持以科学发展观为指导，大力实施“四化两型”战略，积极践行民本岳阳发展理念，加快推进“五市一极”建设和“四三六”工程，圆满完成了本届政府的各项工作任务，在加快富民强市进程中迈出了坚实步伐。

　　——经济发展成效显著。全市GDP跨上2000亿元台阶，由2007年的915.8亿元增加到2012年的2210亿元(预计数，下同)，年均增长13.8%;财政总收入跨上200亿元台阶，由60.8亿元增加到230亿元，年均增长30.5%，占GDP比重由6.6%提高到10.4%;三次产业结构由19.3:48.6:32.1调整为11.6:55.3:33.1。新型工业化加速推进，规模工业增加值年均增长19.4%，巴陵石化和长岭炼化千万吨炼化一体化、华能三期、岳纸40万吨等一批重大项目建成投产，石化产业成为千亿产业，先进制造、节能环保、生物医药、循环经济等产业不断壮大。园区工业增加值年均增长20.7%，占规模工业增加值的比重由39.3%提高到45%，8个工业园区产值均过百亿元，经开区升级为国家级经济技术开发区，汨罗循环经济产业园被列为首批国家“城市矿产”试点示范基地，云溪、平江工业园被列为国家级新型工业化化工新材料产业示范基地、军民结合产业示范基地，中国(湖南)轻工产业园落户湘阴。高新技术产业增加值年均增长37.5%，占规模工业增加值的比重由20.7%提高到32.7%。我市被评为首批全国商标战略示范城市，成功创建国家知识产权试点城市。农业产业化水平提升，农产品加工销售收入年均增长19.4%。粮食生产实现“九连增”，水产品总量连续22年位居全省第一，新型棉花三系不育系在全国率先培育成功。湖南台湾农民创业园落户岳阳县，屈原、华容成为国家现代农业示范区，临湘被列为全国重点产茶县市，我市被授予“中国黄茶之乡”称号。现代旅游业加快发展，建成岳阳楼新景区，岳阳楼—君山岛晋升国家5A级旅游景区，君山被授予“中国野生荷花之乡”和“全国休闲农业与乡村旅游示范区”，旅游总收入年均增长25.6%，占GDP比重由5.6%提高到7.7%。城乡市场繁荣活跃，社会消费品零售总额年均增长19.1%。非公有制经济健康发展，新增市场主体10万户，非公有制经济增加值占GDP比重由51.8%提高到60.3%。县域经济实力明显增强，汨罗、湘阴、华容进入全省经济强县20强，君山进入全省县域经济发展先进县市区。我市被评为新中国成立60年60个城市发展代表，被《福布斯》中文版列为中国大陆最佳商业城市。

　　——城乡面貌明显改观。完成固定资产投资3863亿元，年均增长37.8%。交通建设快速发展，武广客运专线岳阳段、随岳高速公路、通平高速公路和23条干线公路建成通车，岳常、临岳等4条高速公路相继开工;新建千吨级以上泊位17处，新增港口吞吐能力2000万吨。水利投入持续加大，完成水利工程25.3万处，解决农村127.8万人饮水安全，获得“全国节水型社会建设示范市”称号。电力设施不断完善，3座220千伏变电站、18个110千伏输变电项目建成运营，城市居民智能配电网建设稳步推进。城乡生态更加优良，实施减排工程208个，建成运营11座污水处理厂，环南湖截污管网竣工，湘江流域重金属污染治理全面展开，全市森林覆盖率45.3%，空气质量优良率94.6%，岳阳被授予“最值得驻华大使馆向世界推荐的中国生态城市”称号。完成岳阳城市总体规划修编，“五创提质”深入开展，中心城区建成巴陵广场、环南湖旅游走廊千亩湖段等一批标志性工程，拉通了青年路、建湘路等8条“盲肠道”，完成了岳阳大道(西)、巴陵大道等10余条主次干道建筑立面改造。新农村建设扎实开展，新改建农村公路8820公里，100%的乡镇、92.5%的行政村通上水泥路;农村环境整治全面推进，涌现了一大批秀美乡镇、秀美村庄，华容被评为“全国文明县城”，湘阴获评全省城乡环境卫生十佳县。

　　——改革开放深入推进。成功争取678.5平方公里纳入长株潭“两型社会”综合配套改革试验区滨湖示范区，湘阴、汨罗、屈原、临港产业新区四个片区建设扎实推进。临港产业新区完成投资近40亿元，基本实现“三年搭骨架”目标，水电八局机电设备制造岳阳基地、硅峰电动汽车等20个项目竣工投产。城陵矶新港一期建成运营，城陵矶口岸开通海轮航道和“五定”班轮航线，实施“四关联动”通关模式，实现港澳直航常态化。国企改革成效显著，全面完成99家市属国有企业改制任务，3.3万名职工进入社区。市县政府机构改革、乡镇机构改革、财政体制改革、税制改革、集体林权制度改革和医药卫生体制改革实现预期目标。市城建投实现改制转型，新组建港建投、洞庭新城、交建投、水建投等7家投融资平台，逐步形成多元投融资体系。开展城乡建设用地增减挂钩试点，全面推行土地使用权和矿业权网上交易，连续12年实现耕地占补平衡。新增融资性担保公司、小额贷款公司等各类金融机构42家，新增上市公司4家、总量达到10家，9家企业在天津股权交易所挂牌，新设本土创业投资基金2家，市商业银行重组成功，金融机构各项存款余额突破千亿元大关、达到1140亿元，贷款余额达590亿元，岳阳被评为“中国金融生态城市”。对外开放不断扩大，实施内联引资项目2015个，到位资金990.8亿元;利用外资7.8亿美元;引进国际国内500强企业38家。获得进出口权企业达到450家，进出口总额年均增长15%。积极推进长江沿岸、“中三角”、“小三角”城市合作，成功举办北京奥运会火炬传递、第二届湘商大会、纪念平江起义八十周年暨中国(湖南)红色旅游文化节等重大活动;加强与6个国际友好城市交往和经贸合作，经济外向度进一步提高。

　　——社会事业繁荣发展。教育强市建设扎实推进，投入127亿元用于教育事业，各类教育协调发展，我市被评为第三届全国未成年人思想道德建设先进城市。医疗保障水平不断提高，新农合参合率达到99.8%，在全国率先推广“三零”社区卫生服务模式、率先建立地市级造血干细胞配型实验室，在全省率先实现乡镇卫生院、社区卫生服务中心和村卫生室基本药物制度全覆盖，率先建立第三方医疗纠纷调解机制，城市15分钟、农村30分钟医疗卫生服务圈全面形成。全民健身运动蓬勃开展，举办了全省第四届大众运动会和全市第二届大众运动会，市体育中心开工建设，竞技体育水平不断提高。文化建设力度加大，市文化艺术会展中心、美术馆等建成运营，全市图书馆、博物馆、文化馆、纪念馆全部免费开放，城市“十分钟文化圈”基本成形，乡镇文化站实现全覆盖;《今天有客来》、《春雨》荣获“群星奖”金奖，《今上岳阳楼》荣获田汉新剧目奖，《平民领袖》参加“献礼十八大”优秀剧目进京调演，汨罗江畔端午习俗进入世界非物质文化遗产名录。市档案馆晋升国家一级综合档案馆。我市被评为全国文化体制改革工作先进地区和“扫黄打非”工作先进集体。在全国率先开展计划生育手术并发症患者扶助制度试点，计划生育工作连续14年保持全省先进。

　　——人民生活持续改善。全市财政民生支出584亿元，占财政支出的68.5%，省市实事任务全面完成。城镇居民人均可支配收入达到22179元，农民人均纯收入达到8237元，分别净增8576元和3912元。新增城镇就业36.5万人、农村劳动力年均转移就业8.5万人，城镇登记失业率稳控在4.3%以内，屈原成为全省统筹城乡就业示范区试点单位。基本养老、医疗保障实现城乡全覆盖，工伤保险实现职业人群全覆盖，城乡低保实现动态应保尽保，月补差水平分别提高到248元和105元。建设保障性住房5.1万套，实施城市和国有工矿棚户区改造2.5万户，发放廉租住房租赁补贴2.7万户。洞庭湖区捕捞渔民全部上岸定居。在全国率先开展“零就业”家庭就业援助专项行动、率先建立特困家庭大病医疗慈善救助基金;在全省率先实现乡乡都有敬老院。加强食品药品安全监管，社会管理综合治理和安全生产工作连续5年全省先进。岳阳楼区被评为全国和谐社区建设示范城区。

　　——民主法治不断健全。认真落实行政裁量权基准制度和行政执法案例指导制度，严格实施《湖南省行政程序规定》，

　　取消行政审批事项366项，清理规范性文件1180件。加强政务服务中心建设，窗口直接签批率达到95%，审批办理时限压缩30%以上，建成乡镇便民服务中心175个。深入推进廉政建设和反腐败工作，建立健全政府投资项目管理、财政性资金管理、土地管理、规划管理、招投标监督和重大项目监察、审计全程跟踪等制度，严查了一批渎职贪腐案件。自觉接受人大法律监督和政协民主监督，承办人大议案、代表建议和政协建议案、委员提案3361件。顺利完成村、社区换届选举，基层自治机制不断完善。工会、共青团、妇联等群团组织的桥梁和纽带作用充分发挥。

　　2012年是本届政府任期的最后一年。一年来，我们牢牢把握“稳中求进、进中求强”的工作总基调，全面推进“四三六”工程，加强经济形势研判和经济运行调度，保持了经济平稳较快发展。预计GDP增长12%，财政总收入增长23.8%，固定资产投资增长35.5%，社会消费品零售总额增长15.2%，城乡居民人均收入分别增长13.4%和16.5%，人口自然增长率5.74‰，居民消费价格指数涨幅2.1%，万元GDP能耗下降6%，超额完成节能减排任务。这一年，我们突出抓了五个方面工作。

　　突出宏观长远谋划。谋划和推进洞庭湖生态经济区建设。岳阳市域“1+9”规划编制工作基本完成，初步确定“十大工程、千个项目、万亿投资”的发展目标，158个项目纳入省规划项目库。谋划和推进湖南长江经济带建设。重点抓好现代航运物流业、湘北能源基地、湖南岳阳绿色化工产业园、现代旅游服务业、现代农业的发展，加快构建具有沿江特色的产业体系，正在努力争取成为全省区域重点发展战略。临港产业新区获批省级高新技术产业开发区，申报国家综合保税区、进口肉类指定口岸、汽车整车进口口岸工作进入国务院审批流程;湖南岳阳绿色化工产业园规划获省政府批复，巴陵石化20万吨己内酰胺挖潜改造等5个项目竣工投产，中石化催化剂云溪基地一期竣工运营;湖南电煤储备基地前期工作加速推进。谋划和推进城市长远发展。规划建设环南湖片区，推行“三统一、一共享”模式，致力打造现代滨湖新城区、旅游休闲度假区、生态文化新景区;启动调整东洞庭湖自然保护区规划，拓展城市发展空间;加快推进洞庭新城建设，提升城市生活品质;实施“四湖两河”连通工程，凸显城市生态宜居特色。

　　突出项目引进建设。扎实开展项目引进和建设攻坚行动，100个重大建设项目99个开工，完成投资244.9亿元;82个重大前期项目开工18个。湖南科伦一期工程、中科产业园、远大可建、远大住工、福坤汽车产业园一期工程等项目竣工投产;京港澳高速公路连接线改造升级、中心城区37条主次干道油化提质和12条道路绿化提质等项目全面完成，城区青年东路、德胜北路全线拉通;洞庭湖综合治理、环洞庭湖基本农田重大建设工程、芙蓉大道北拓湘阴段、炼化一体化公路、S207平江段、岳宜高速公路等项目加快建设;荆岳铁路、临湖公路、岳望高速等项目启动开工。积极参加“中博会”、“产洽会”，成功举办第九届中国城市友好商会经济协作会暨百强民营企业岳阳行、中国(岳阳)首届工业磁力及熔炼技术装备展览会等活动，成功申办2013年中国茶叶经济年会暨岳阳茶叶博览会。中联重科临港工业园、中石化和壳牌中国公司第二条煤气化生产线、塞拉维电动汽车、南翔万商岳阳国际商贸物流城等一批大项目签约落户。共实施内联引资项目657个，到位资金304.8亿元;引进外资2.4亿美元，增长29%。

　　突出产业转型升级。强力推进节能减排淘汰落后产能攻坚行动，拆除和停产35条生产线，关停54家“十五小”企业，城区首批“退二进三”企业5家停产、4家落户园区。高新技术产业加快发展，高新技术产业增加值增长38%，生物医药、电子光伏等新兴产业分别增长25%和29%，全国首个磁力行业标准化工作组在岳成立，国家磁力设备质量监督检验中心加快建设。与上海、广州、成都一道获批北斗应用示范城市和北斗导航营运中心。全面启动湘江航道岳阳段疏浚工程，开通港澳直达航线，投入运营联检锚地，集装箱吞吐量达到19万标箱，港口货物吞吐量突破亿吨大关，岳阳成为长江沿岸亿吨级大港城市。现代旅游业持续升温，左宗棠文化园、君山野生荷花世界等项目竣工运营，屈子文化园、张谷英引景区、洋沙湖度假区等项目加快建设，成功举办第八届汨罗江国际龙舟节、第七届洞庭湖国际观鸟节、首届荷乡美食文化节、纪念左宗棠诞辰200周年等节会，国家级洞庭湖旅游度假区建设通过省级验收，全年接待游客1680万人次，实现旅游总收入170亿元。园区工业提质发展，华容、岳阳县工业园获批省级工业集中区，湘阴工业园获批湖南省电子信息产业示范基地。实施品牌战略，设立“市长质量奖”，新增巴陵春、兰岭、宏岳、滨湖4件中国驰名商标。

　　突出优化发展环境。全面开展“五创提质”活动，创建全国绿化模范城市顺利通过国检，国家卫生城市顺利通过复检，全国城市文明程度指数测评进入提名资格城市前11强，未成年人思想道德建设工作位列全国地级城市前50强。大力实施城乡环境整治攻坚行动，王家河流域综合治理、铁山水库水资源保护、环南湖亮化提质改造、城区严重渍水地段改造等工作进展顺利，城乡环境卫生面貌焕然一新，市爱卫办被评为全国爱国卫生工作先进单位，农村“清洁家园”行动排名全省第一。强力推进禁违拆违治违行动，拆除违法建设9.5万平方米，新的违法建设基本控住。扎实推进“洞庭风雷”行动，严厉打击非法采砂、非法捕捞、非法排污、非法水上营运，开展洞庭湖水生生物增殖放流活动，洞庭湖水环境与水资源得到有效保护。积极开展“文明餐桌”行动，“文明用餐、节俭惜福”成为新风尚。深入开展“四化六评”、“五个一”和重点工程建设环境集中整治等优化经济环境活动，12345政府服务热线被评为全国优秀服务热线。组织开展“下基层、访民情、化积案、办实事”、“百个部门联社区、万名干部进家庭”等专项活动，社会管理综合治理考核全省排名第二，我市被评为全国信访工作先进集体。

　　突出保障改善民生。全年财政民生支出165亿元，占财政支出的70%。新增城镇就业人员5.5万人、农村劳动力转移就业7.1万人;开工保障性住房建设和各类棚户区改造41285套，排名全省第一。企业养老保险、城镇职工医保、失业保险、工伤保险、生育保险覆盖率稳步提高;城镇职工、居民医保住院报销率分别提高至78.2%和70.3%。新农合医疗住院费用可报比例达到90.5%，排名全省第一，临湘、平江在全省率先推行新农合乡镇首诊全免费，屈原在全省率先实现医疗保险城乡统筹，岳阳楼区成功创建国家慢性病综合防控示范区。出台《岳阳市贫困重度残疾人特殊生活补贴实施办法》，3.3万名贫困重度残疾人纳入补贴范围。建设校安工程60个、义务教育合格学校93所、公办幼儿园42所，中心城区“阳光招生”顺利实施。市广电中心开工建设，博物馆改造、广播电视“村村通”等文化项目加快建设，新建1076个农家书屋，提前实现村村有农家书屋的目标。成功举办第四届湖南艺术节活动和长江流域八省市老年书画联展。

　　其他各项事业加快发展，国防动员、国安、人防、统计、史志、保密、老干老龄、民族、宗教、社会科学、科协、气象、对台、驻外联络、住房公积金、移民、烟草、石油、邮政、通信、盐业等工作取得新进步。

　　各位代表，回顾过去五年的工作，成绩来之不易，奋斗尤为艰辛，经验弥足珍贵。这些成绩的取得，是省委省政府正确领导、关怀支持的结果;是全市人民团结奋斗、顽强拼搏的结果。在此，我代表市人民政府，向全市人民，向一切关心、支持和参与岳阳建设发展的社会各界人士，表示衷心的感谢!

　　同时，我们也清醒认识到，我市经济社会发展还存在许多不足和困难。主要是：产业结构、财税结构不优;研发投入不够，创新能力不强;城乡居民收入差距较大;社会管理和公共服务依然薄弱，保障和改善民生任务繁重;资源环境约束趋紧，节能减排压力很大;政府自身建设需要继续加强，发展环境有待进一步优化。对这些问题，我们将采取有效措施，认真加以解决。

　　今后五年目标任务和2013年工作

　　未来五年是加快富民强市，建设美丽岳阳、幸福岳阳的关键时期。党的十八大提出了全面建成小康社会和全面深化改革开放的目标，描绘了小康社会的美好前景。国家继续实施中部崛起战略，长株潭“两型社会”建设、洞庭湖生态经济区、湖南长江经济带建设加快推进。岳阳经济社会发展中的积极因素、竞争优势累积叠加，发展基础更加稳固，发展后劲不断增强。同时，我们也面临着经济增速放缓、区域竞争加剧、转型升级压力加大、改善民生任务加重等诸多困难。面对新的机遇和挑战，面对岳阳人民过上更好生活的新期待，我们要切实肩负起时代和人民赋予的重任，抢抓机遇，凝聚力量，攻坚克难，奋力开创岳阳全面建成小康社会的新局面。

　　今后五年的工作思路是：认真贯彻落实党的十八大精神，坚持以科学发展观为指导，紧紧围绕主题主线，着力提高经济增长质量和效益，积极实施“四化两型”战略，深入推进“五市一极”建设和“四三六”工程，深化改革开放，促进产业升级，创新社会管理，保障改善民生，加快建设宜业宜游宜居的美丽岳阳、幸福岳阳，努力走在全省全面建成小康社会的前列。

　　今后五年的奋斗目标是：

　　实现 “两个提前”——提前到2015年实现GDP、财政总收入和城乡居民人均收入比2010年翻一番;提前到2018年全面建成小康社会。

　　迈上“两个台阶”——到2017年，GDP迈上4000亿元台阶，达到4110亿元，年均增长10%以上;财政总收入迈上400亿元台阶，达到440亿元，年均增长14%以上。

　　建设宜业宜游宜居的美丽岳阳、幸福岳阳。

　　营造政策优惠、服务优质、公平公正的创业环境，激发全社会创业创新创造活力，形成就业渠道广、创业机会多、产业发展旺的生动局面，致力建设宜业岳阳。营造设施完善、要素齐备、人气兴旺、独具特色和魅力的旅游休闲环境，促进岳阳由旅游过境地转变为目的地，致力建设宜游岳阳。营造社会文明、生态秀美、生活便利、安全有序的工作生活环境，提高人民幸福指数，致力建设宜居岳阳。

　　2013年是深入贯彻落实党的十八大精神的开局之年，是新一届政府的起步之年，是实施“十二五”规划承前启后的关键之年，是建市30周年的喜庆之年。做好2013年工作，必须深入贯彻落实中央、省委、市委经济工作会议精神，以“四三六”工程总揽工作全局，实施沿江开放发展战略、新型城镇发展战略、绿色生态发展战略、改革创新发展战略，做好湖南长江经济带、洞庭湖旅游度假区、县域经济、战略性新兴产业四篇文章;加力项目引进与项目建设攻坚、城乡环境整治攻坚、节能减排与淘汰落后产能攻坚，提升产业层次、城镇品质、园区实力，实现经济社会发展的良好开局，为全面建成小康社会打下坚实基础。2013年经济社会发展预期目标是：GDP增长11%以上;财政总收入增长16%以上，规模工业增加值增长16%以上，固定资产投资增长23%以上，社会消费品零售总额增长16%以上，城乡居民人均收入分别增长12%和13%以上，居民消费价格指数涨幅控制在3%左右，城镇登记失业率控制在4.3%以内，人口自然增长率控制在6.6‰以内，全面完成省下达的节能减排任务，各项社会事业都有新的进步。

　　今后五年和2013年工作的主要任务是：

　　一、转变发展方式，推动经济持续健康发展

　　坚持把转变经济发展方式作为全局性、战略性任务来抓，努力走出一条符合岳阳实际的转型发展、科学发展道路。

　　坚持两型引领，构建现代产业体系。实施改革创新发展战略，加快建设创新型岳阳，着力打造一批企业技术创新平台，促进产业转型升级，力争研发经费支出占GDP比重达到2.5%。加速推进新型工业化，改造提升石化、食品、纺织、建材等传统优势产业，加快发展电子信息、现代装备制造、生物医药等战略性新兴产业，创建一批信息化示范企业，推进信息化与工业化深度融合。突出抓好长岭炼化1150万吨炼油改扩建、巴陵石化350万吨炼油和非乙烯精细化工系列改造、岳纸70万吨涂布包装纸技改、中轻国泰大型造纸装备机械国产化基地、中国(湖南)轻工产业园、中粮集团岳阳粮油产业园等一批优势产业项目;突出抓好国信军创北斗导航产业岳阳基地、中联重科临港工业园、湖南科伦生物医药园、塞拉维电动汽车产业园、福坤汽车产业园等一批新兴产业项目。优先发展现代物流、金融保险、中介服务等生产性服务业，全面提升商贸流通、餐饮住宿、体育健身、休闲娱乐、家政养老等生活性服务业，积极发展文化、创意、旅游等特色服务业，加快发展总部经济、楼宇经济和电子商务，力争第三产业增加值占GDP比重达到50%。加大节能减排力度，实施十大环保工程，推进清洁生产和资源综合利用，加快火电机组脱硫脱硝工程、城镇污水处理和垃圾处理设施等项目建设，万元GDP能耗年均下降6.9%。强化用地保障，加强闲置土地清理处置和土地批后监管，提高土地节约集约利用水平。推进长株潭“两型社会”建设滨湖示范区第二阶段建设，力争在两型产业发展上取得新进展。

　　发挥港口优势，打造湖南长江经济带。实施沿江开放发展战略，大力发展现代航运物流业，围绕“百万标箱、亿吨大港、千亿园区”的目标，高标准修编《城陵矶新港区总体发展规划》，完成城陵矶新港二期建设，推进沿江港口群建设和沿江产业集群发展，力争临港产业新区成功创建国家级高新区，建设成为全省综合交通运输体系的重要枢纽、现代物流的重要园区、开放型经济的重要门户。2013年完成临港产业新区核心区路网及配套工程、标准化厂房二期工程，推进口岸联检大楼改造升级、电子口岸平台建设，提高通关效率，集装箱吞吐量达到22万标箱，力争国家综合保税区、进口肉类指定口岸、汽车整车进口口岸申报工作取得重大突破。加快推进湖南岳阳绿色化工产业园建设，以长岭炼化、巴陵石化为龙头，以长炼工业园、临港产业新区化工新材料园、云溪精细化工产业园、临湘滨江产业示范区为平台，延伸壮大四条产业链，实现原油加工总量达到1500万吨、产值达到2000亿元，力争成功创建国家级绿色化工园区。建设好湘北能源基地，加快荆岳铁路松阳湖支线建设，建成湖南电煤储备基地，推进沿江沿湖风力发电项目建设，继续做好华容小墨山核电、平江抽水蓄能发电等项目前期工作。

　　依托楼岛湖江资源，做强“花、鸟、舟、情”品牌。以岳阳楼—君山岛5A级景区为龙头，整合旅游资源，大力开发参与型、娱乐型、文化主题型旅游产品，完成洞庭湖国际公馆、洞庭湖博物馆、旅游实景剧等重点项目建设，促进旅游业由观光型向观光休闲度假复合型转变，成功创建国家级洞庭湖旅游度假区。充分挖掘君山团湖莲(廉)文化、洞庭湖湿地生态文化、汨罗江龙舟文化、君山岛爱情文化等特色资源，运用市场手段办好君山团湖野生荷花节、洞庭湖国际观鸟节、汨罗江国际龙舟节和君山岛爱情主题活动，做强“花、鸟、舟、情”特色旅游品牌。加快县域特色旅游开发，着力发展运动休闲、民俗古韵、红色文化、生态度假等旅游板块，完成屈子文化园、任弼时纪念馆二期、左宗棠文化园二期、石牛寨国家级地质公园、张谷英引景区、洋沙湖度假村、濠河城市湿地公园等项目建设，提升县市区旅游产业品质。创新营销方式，加强长江沿线城市旅游线路合作，开发“岳阳—赤壁·穿越江湖”水上观光游览线路;利用京广高铁全线开通，积极开拓北方新兴客源市场，丰富旅游精品线路。2013年办好建市30周年庆典、中国十大经典爱情故事评选颁奖、中国茶叶经济年会暨岳阳茶叶博览会等节庆活动，带动购物、文化、娱乐、餐饮等旅游消费升温，促进旅游产业全面发展，把岳阳建设成为令人向往流连的旅游目的地。

　　立足园区升级，提升县域经济实力。加强园区规划引导，推动要素向园区聚集、企业向园区布局，促进园区发展转型升级，着力提升园区实力。推进经开区“二次创业”，重点打造亚洲工业磁力应用设备制造基地、全国造纸装备自主研制基地、生物医药产业基地、北斗卫星导航产业应用示范和营运基地。加快汨罗“城市矿产”基地、湘阴先进装备制造和电子信息产业园、临湘滨江产业示范区、平江汽车和工程机械零部件基地、华容医药纺织和电子光伏产业园、岳阳县生物医药建材和粮食加工产业园等园区建设，构建产业各异、功能完善、分工协作的园区体系。以工业园区为载体，大力发展特色经济、非公有制经济、开放型经济，辐射带动县域经济全面发展，争取更多的县市区进入全省县域经济10强、20强。

　　二、统筹城乡发展，加快城乡一体化进程

　　把解决农业、农村、农民问题作为政府工作重中之重，坚持以工促农、以城带乡、城乡互动，加大统筹城乡发展力度，增强农村发展活力，促进城乡共同繁荣。

　　加强“三农”工作。稳定发展粮食生产，加强耕地保护和农村土地综合整治，继续推进环洞庭湖基本农田重大建设工程，确保全市粮食播种面积稳定在800万亩以上，建成洞庭湖国家粮食安全保障区。推进现代农业发展，壮大蔬菜、棉花、油菜、茶叶、水产品等优势产业，扩大畜禽水产规模化、标准化养殖，抓好临湘楠竹、平江油茶科技示范点建设，大力发展林下经济。加快屈原和华容现代农业示范区、岳阳县湖南台湾农民创业园、君山全国绿色食品建设示范基地和黄茶产业园建设;积极发展农产品加工园区和农副产品物流园区，继续实施“1351”龙头企业发展计划，2013年实现食品产业产值过千亿元。加快农村现代流通网络体系建设，大力发展农民专业合作社，做大做强宏岳科技期货物流园、海泰食品冷链物流园。加强农业科技创新，推广应用优良品种和高产、高效绿色环保技术，提升农业机械化水平，强化农产品全程质量监管。坚持“办点示范、点面结合、成片推进”的原则，抓好新农村建设城乡统筹试点镇和示范创建片(村)建设。深入实施农村“清洁家园”行动，加快推进农村水、电、路、桥、通讯等基础设施建设，突出抓好河道采砂整治、地质灾害防治、农村面源污染治理，组织开展秀美县城、秀美乡镇、秀美村庄和星级文明农户创建活动。加强农村文化建设和农民技能培训，提高农民素质，充分发挥农民在新农村建设中的主体作用。

　　推进新型城镇化。实施新型城镇发展战略，构建“一核三圈”城镇格局，推进规划同编、设施同建、交通同网、环境同治、产业同兴，力争全市城镇化率达到60%、地区经济发展差异系数控制在60以下。湘阴、汨罗、平江和屈原加快对接长株潭;临湘、华容加快建设成为省际边界重点城市;推进云溪城南新区开发和岳阳县荣湾湖综合治理，加快与中心城区对接步伐;实施跨湖发展战略，促进君山建设成为沿江环湖洞庭生态明珠;加快岳阳楼区梅溪、七里山、洛王、郭镇片区建设，实现同城一体化发展。完善城镇体系规划和乡村规划编制，加快城镇基础设施建设，健全城镇公共服务体系，鼓励农村劳动力就近向城镇转移，引导乡村居民点集中布局，有序推进农业转移人口市民化，着力提升城镇品质。大力发展园区型、边贸型、旅游型、休闲度假型等特色城镇，2013年创建10个省级中心镇，重点扶持20个特色镇发展。

　　加快基础设施建设。交通：建设岳阳机场，2013年完成立项审批工作;完成岳常、岳望、岳宜、临岳4条高速公路和京港澳高速岳阳收费站西移改造、芙蓉大道北拓湘阴段等项目建设，力争平益高速开工建设;建成荆岳铁路，启动岳长、长平汨城际铁路和岳吉铁路建设，做好岳九、岳常铁路前期工作;加大S207平江段等国、省道公路升级改造和养护力度，新增二级以上干线公路600公里;启动农村公路连通工程，实现所有具备条件的建制村通水泥(沥青)路;加快站场建设，构建多种运输方式高效衔接的综合枢纽。水利：加快防汛抗旱减灾工程、农田水利建设工程、城乡供水工程、水生态保护工程、农村河流治理工程、水土保持工程等重大民生水利工程建设，加快推进城陵矶水利综合枢纽工程前期工作，争取启动汨罗江、华容河、黄盖湖综合治理和三峡后续影响处理等重大项目，改扩建中央防汛抗旱物资储备中心。2013年完成城西垸、屈原垸围堤加固工程，新墙河、游港河等9条中小河流治理，5个县市区小农水重点县建设，256座病险水库除险加固工程，解决农村30万人安全饮水问题。电力：加快输变电设施建设，完成农村电网改造升级，完善城乡输配电网体系;支持国电、华能、神华加速推进4×100万千瓦火电项目建设。

　　扩大城乡居民消费。大力发展汴河街、东茅岭商业街、湖鲜美食街等特色街区，支持天伦城、步步高、人和春天等商业街区建设和发展，做强传统特色商业板块，建设城市核心商圈。加快金凤桥片区开发、南翔万商岳阳国际商贸物流城建设，规划建设好天灯咀南湖水商业街，推进云溪、挂口、城陵矶、路口、湖滨、八字门等区级商业区建设，形成多层次城市商业网点体系。优化住房供应结构，合理引导住房消费，促进房地产业健康稳步发展。积极落实“节能家电推广”等消费政策，推进“万村千乡”市场工程，重点扶持十三村、和田等本土流通企业发展农家店，支持农村特色种养、休闲农庄发展，活跃农村消费市场。

　　三、彰显山水特色，建设水墨丹青岳阳

　　在推进城市建设和发展中，始终突出山水特色、生态优势，实施绿色生态发展战略，使岳阳处处鸟语花香、郁郁葱葱，成为一幅山水相融、城水相依、人水相亲的水墨丹青。

　　实施提质扩城，让岳阳更大气。重点以临港产业新区和经开区建设推动城市北靠东扩，以环南湖片区建设加快城市南延，以推进跨湖发展促进城市西连，以洞庭新城建设带动旧城改造，提升中心城区品质，加快建成100万以上人口的特大城市。完善规划体系，严格规划管控，推进城区“三纵八横”路网和临湖路等环城道路建设，加快城市供排水系统、污水处理系统、垃圾储运网络、公交专用道路和站场建设，新建大桥河、珍珠山、架子山、天灯咀和龙山公园等景区景点。推进城区综合管沟建设，开发、经营、管理好城市地下空间资源。突出抓好重点区域、重点地段的城市形象和景观设计，开展城区“十大精品建筑”评选活动，建成一批具有岳阳特色的标志性建筑、城市广场和雕塑。2013年完成洞庭南路、桂花园路提质改造和岳城路等10条城市支路建设，建成市妇幼保健院路段人行过街设施和桥西、东风广场等10处小游园;完成城区严重渍水地段整治改造和农贸市场、公厕垃圾站、环南湖及城市主次干道亮化提质改造;启动枫桥湖路3517段等六路建设和花板桥等立体交通工程、三大湖滨水休闲景观工程和湖滨、马壕污水处理厂建设。

　　实施森林进城，让岳阳更清新。深入推进绿色城市、绿色屏障、绿色村镇、绿色通道工程，继续开展城区主次干道绿化提质行动，加快形成景园均衡布局、环城绿荫围绕、道路绿带相依的城市绿化景观格局，力争2017年城市建成区绿化覆盖率43%、绿地率40%。加强福寿山风景名胜区和幕阜山、大云山、五尖山、桃花山、玉池山、天井山等国家、省级森林公园保护开发，加强森林资源管护和湿地保护，大力实施沿江、沿河、沿湖、沿路绿化工程，推进生态移民工程，巩固退耕还林成果。加强PM2.5监测治理，严格污染物总量控制，力争环境质量指数达到100%，使岳阳天更蓝、地更绿、水更清。

　　实施流水环城，让岳阳更灵秀。以“水清、水活、水利、水美”为目标，大力推进“四湖两河”连通工程，完成王家河流域综合治理工程，打造城市生态景观带和滨水文化长廊;加强南湖、东风湖、芭蕉湖、吉家湖、北港河等水系保护和治理，恢复原有水体轮廓线，建设亲水型城市。推进洞庭湖沿湖风光带与环南湖旅游走廊融合对接，建设环南湖旅游走廊机动车道、国际自行车赛道、游人步道等交通三圈，让南湖“运动”起来。扎实推进洞庭湖综合治理、湘江流域重金属污染治理、松阳湖等水污染综合整治，推进沿湖沿河污染企业“退二进三”、搬迁入园，加强洞庭湖水生生物和水环境保护，完成江豚保护区建设，构建人水和谐的生态环境。2013年继续开展“洞庭风雷”行动和河道采砂专项整治，促进河湖安澜。实行最严格的水资源管理制度，加强铁山水库水资源保护，坚持“禁、关、拆、补”综合治理，让市民群众一直喝上放心的铁山水。

　　实施精细管城，让岳阳更宜居。加强城市环境卫生、市容秩序、市政设施等管理，创新城市管理投入、巡查、考核、奖惩机制，促进城市管理信息化、标准化、网格化、常态化。推进“智慧岳阳”工程，抓好智慧交通、数字城管、平安岳阳建设，加快三网融合和物联网建设，2013年完成城市治安电子防控系统三期工程。深入开展交通秩序、卫生环境、农集贸市场、“五小”门店、人行道板“五大整治”行动，巩固提升国家卫生城市创建成果。坚持疏堵结合、城乡联动，以铁的手腕、铁的纪律坚决拆除存量违法建设，坚决禁住新增违法建设，坚决查处搞违法建设的人和事。继续推进“五创提质”，巩固绿化创模成果，力争2013年成功创建国家交通管理模范城市、国家环境保护模范城市、国家社会管理综合治理先进市，2014年成功创建全国文明城市，让城市更安全、更文明、更舒适，提高市民对城市的归属感、认同感和自豪感。

　　四、深化改革开放，增强发展动力和活力

　　坚持以更大的决心和魄力推进改革开放，着力激发各类市场主体发展新活力，着力培育开放型经济发展新优势。

　　加快改革步伐。深化经济体制改革，支持引导非公有制经济加快发展，力争非公有制经济增加值占GDP比重达到66%。引进兴业、招商、中信等股份制商业银行，积极发展村镇银行，扶持发展融资性担保公司、小额贷款公司等地方金融机构，加快推进农村信用社改革，规范搞活民间资本，金融机构存贷比突破60%。切实改善金融生态，扩大直接融资，培育后备资源，争取更多的企业成功上市。规范完善投融资平台管理，建立现代企业经营管理制度，加大国有资本在公益事业、民生领域的投入力度，增强国有资本的保值增值能力。推进财政预算绩效管理，深化财税管理改革，健全公共财政体系。深化公共服务改革，推进文化体制、医药卫生体制、水利体制、户籍制度和殡葬改革，构建比较完备的基本公共服务体系。深化农村综合配套改革，继续抓好土地流转、征地制度和农村投融资改革等，增强农村发展活力。深化行政体制改革，严格控制机构编制，加快推进事业单位分类改革和公车改革，促进政府职能转变和行政效能提升。2013年扎实开展第三次全国经济普查，积极引导个体工商户向法人企业转型。　加强区域合作。以临港产业新区为平台，主动融入“两带”、“两圈”，进一步扩大开放领域，拓展发展空间。以洞庭湖生态经济区为依托，加强与长沙、益阳、常德等地合作，突出规划、交通、产业、旅游、市场对接，促进要素资源合理配置，实现优势互补、融合发展。以岳咸九“小三角”战略联盟为契机，加强交流合作，打造长江中游城市合作示范区。以湘鄂赣边界23个集镇为基础，扩大边贸往来，繁荣边贸经济。

　　发展外向经济。发挥城陵矶口岸优势，大力实施进出口“百、千、亿”发展计划，加大国际商标注册力度，努力扩大农产品、机电产品出口总量和劳务输出规模，不断提高经济外向度。以更大力度、更高水平抓好招商引资，创新招商方式，突出招大引强，主动对接国内外战略投资者，积极承接长三角、珠三角产业转移，着力引进一批大项目、好项目，切实提高引进资金到位率、项目开工率和投产率。

　　五、发展社会事业，保障和改善民生

　　坚持把保障和改善民生作为政府一切工作的出发点和落脚点，认真落实惠民利民政策，多谋民生之利，多解民生之忧，努力让全市人民过上更好生活。

　　积极扩大就业。实施更加积极的就业政策，多渠道增加就业岗位，重点做好高校毕业生、城镇困难人员、失地农民等就业工作，城镇登记失业率控制在4.3%以内，新增农村劳动力转移就业20万人以上，城镇“零就业”家庭保持动态清零。加快创业型城市建设，支持屈原管理区创建湖南省统筹城乡就业示范区。加强劳动保障监察和争议调解仲裁，构建和谐劳动关系。

　　增加居民收入。逐步提高并严格执行最低工资标准，建立工资稳步增长机制，全面推行企业工资集体协商，增加居民工资性收入;深化全民创业行动，增加居民经营性收入;引导居民投资实体经济，增加居民财产性收入。推动居民收入增长和经济发展同步，逐步缩小城乡差距，城乡居民收入比降低到2.8以下。深入推进以平江县为重点的扶贫攻坚行动，增强贫困地区造血功能，加快贫困群众脱贫致富，使贫困地区人民共同奔向全面小康。

　　提高健康水平。优化中心城区医疗布局，积极整合医疗资源，加快建设市中心医院，推进市公共卫生应急救援指挥中心、妇幼保健院、4个县人民(中)医院、55个乡镇(中心)卫生院新改建等项目建设，形成布局合理、功能完善的城乡医疗服务体系，着力缓解看病难、看病贵的问题。深化公立医院改革，推动医药分开，鼓励社会办医。健全全民医保体系，完善医患纠纷第三方调处机制，加强突发公共卫生事件应急管理。开展爱国卫生运动和全民健身运动，加强血吸虫病防治，促进人民身心健康，力争城乡居民平均预期寿命达到75岁。完成市体育中心建设，办好湖南省第八届少数民族传统体育运动会。坚持计划生育基本国策，完善利益导向机制，提高出生人口素质，稳定低生育水平，统筹解决人口问题。

　　健全保障体系。推进社会保险提标扩面，基本社会保险覆盖率达到90%以上。加快保障性安居工程建设，推进住房公积金支持保障房建设试点项目，2013年完成保障性住房建设和各类棚户区改造2.5万套，改造农村危房5000户。落实五保供养、城乡低保、大病救助、重度贫困残疾人救助等扶持政策，切实做好留守儿童、残疾人、孤儿、流浪乞讨人员等弱势群体的救助管理工作，加快残疾人康复托养事业发展。稳控物价水平，推进价格惠民，完善社会救助和保障标准与物价上涨挂钩的联动机制，保障困难群众基本生活。认真落实“菜篮子”市长负责制，支持君山无公害蔬菜基地建设。做好老干老龄工作，鼓励发展社会养老事业，完善居家、社区、机构养老服务体系，推进市老年活动中心、福利院老年福利大楼建设，实现老有所养、老有所乐、老有所为。　 发展教育事业。坚持教育优先，加大教育投入，深化基础教育课程改革，提高教师师德水平和业务能力，促进各类教育协调发展，努力办好人民满意的教育，力争新增劳动力平均受教育年限达13年以上。优化教育资源布局，加强校安工程建设，2013年全市建成50所义务教育合格学校、52所农村公办中心幼儿园。继续实施“阳光招生”政策，让每一个孩子都沐浴到教育公平的阳光。推进人才强市战略，实施“312”工程，加大科技创新人才的引进、培养和奖励力度，加强人才服务工作，激发人才创造活力，营造人尽其才、才尽其用的良好环境。

　　建设文化强市。积极践行社会主义核心价值观，加强社会公德、职业道德、家庭美德、个人品德教育，提高社会文明程度，弘扬时代新风。鼓励文化艺术创作，进一步打响“文坛岳家军”品牌。实施文化强市十大工程，推进市广电中心、青少年活动中心、图书馆新馆、档案馆等文化惠民项目建设，基本建成覆盖城乡的四级公共文化服务体系，成功创建国家公共文化服务示范区。加快发展文化产业，力争文化创意产业增加值占GDP比重达到6.5%。

　　六、创新社会管理，营造和谐发展环境

　　加快推进社会体制改革，提高社会管理科学化水平，努力开创社会和谐人人有责、和谐社会人人共享的生动局面。

　　推进依法治市。认真实施《法治湖南建设纲要》，加强普法教育和政府法制工作，推进行政执法主体和行政执法人员资格认证制度，规范行政权力运行，提高全社会法治水平，建设法治岳阳。加强民主政治建设，自觉接受人大法律监督和政协民主监督。扩大基层民主，完善政务、厂务、村务公开，健全城乡基层群众自治机制。加强国防动员和后备力量建设，深入开展双拥共建，巩固发展军政军民团结。

　　加强社区建设。按照“社会管理社区化，社区管理网格化”的要求，加快城镇社区建设，创新社区管理体制，改善社区办公条件，加大社区基础设施投入，提高社区服务功能。中心城区社区办公用房全部达标，2013年完成20个社区办公用房建设。以村民自治为重点，加快农村社区建设，深化村级民主管理，建设公共服务完善、村容村貌良好的新型农村社区。建立全市人口基础信息库，推行社区流动人口居住证一卡通制度，实现流动人口管理全覆盖。引导社会组织健康有序发展，充分发挥群众参与社区管理的基础作用。

　　严格市场监管。实施更加严格的市场准入制度，建立食品药品安全风险预警机制和责任追溯体系，加强社会诚信建设和消费者维权工作，提高市场监管能力，严厉打击商业欺诈、制假售假等不法行为，扎实开展餐饮宾馆、学校食堂、建筑工地、农村市场等重点领域食品安全专项整治行动，让人民群众安全消费、大胆消费，真正吃上放心菜、放心油、放心食品。

　　强化安全生产。全面落实安全生产“一岗双责”、工作督查约谈制度，加强安全生产审批和验收，积极开展企业安全生产标准化建设和安全生产示范乡镇创建活动，扎实推进打非治违行动，加大非煤矿山、道路交通、水上安全、建筑施工、特种设备、消防安全、危化物品、烟花爆竹等重点行业的专项整治，完善公共安全预防预警和应急处置体系，坚决防止重特大安全事故发生。

　　维护社会稳定。坚持以法治思维和法治方式化解社会矛盾，实行重大决策、重大项目信访稳定风险评估制度，依法规范和治理信访秩序，深入开展领导干部接访约访下访、部门单位联合接访活动，加强人民调解、行政调解、司法调解，切实解决群众合理诉求。加强社会治安防控体系建设，完善县乡村三级综治维稳平台和社会治安联防联控机制，严厉打击违法犯罪行为，加强对刑释解教人员、社区矫正对象、精神病患者等特殊人群的服务管理。加强网络社会管理，唱响网上主旋律，健全新闻发布会、舆情应对制度，提高舆情引导处置能力，维护岳阳政通人和的良好局面。

　　努力建设人民满意政府

　　完成今后任务，做好当前工作，必须严格按照中央改进工作作风、密切联系群众的八项规定，加强政府自身建设，努力建设一支高素质的公务员队伍，努力建设一个人民满意的政府。　 建设责任政府。强化责任意识，夙夜在公、勤勉工作，激发干事成事的激情，保持攻坚克难的锐气，敢于担当责任，敢于面对矛盾，敢于应对挑战。全面落实岗位责任制、首问负责制、服务承诺制、限时办结制，实行更加严格的目标管理、绩效考核、督查督办和无为问责，使政府公职人员人人有责、事事担责。以对岳阳长远发展、对岳阳人民高度负责的精神，多干打基础、利长远的实事，多办惠民生、得民心的好事，不搞短期行为，不留发展后患，努力追求没有水分的增长、真金白银的发展、群众满意的政绩。　 建设服务政府。坚持改进作风，深入开展“机关作风建设年”活动，力戒形式主义和官僚主义，讲真话，报实情，不做假，不作秀，开短会、讲短话、发短文，减少各类一般性检查、考核、评比和庆典活动，减少迎来送往和各种应酬，把更多的精力投入到抓发展和为民服务上。坚持令行禁止，克服办事拖沓、推诿扯皮现象，出台的制度和规定，要求群众做的，政府首先做到;要求群众不做的，政府首先不做;说了的坚决做到，定了的坚决执行。坚持狠抓落实，防止以会议落实会议、以文件落实文件，看准的事情，就要坚持不懈，紧抓不放;部署的工作，就要雷厉风行，真抓实干，有督查、有讲评、有奖惩，一级抓一级，一级对一级负责，咬定青山不放松，不达目的不罢休。

　　建设法治政府。认真落实市人大及其常委会的决议决定，定期报告重要工作，支持和保障人大代表依法履行职责。健全协商民主制度，积极支持市政协履行政治协商、民主监督、参政议政职能，充分听取各民主党派、工商联、人民团体的意见。健全政府工作集体决策、专家咨询、公开听证等制度，推进网络问政，确保每一个重大决策、每一项行政行为都遵循法治的轨道、符合人民的意愿，确保政府工作步调一致、高效运转。深入实施《湖南省行政程序规定》，坚持依法行政、文明执法，着力解决有法不依、执法不公等群众反映强烈的突出问题，以政府依法行政带动全社会的知法守法，以政府诚信带动全社会的诚实守信，让生活在岳阳这片土地上的人们处处感受到民主的光辉、法治的力量、正义的温暖。

　　建设清廉政府。坚持不懈地开展廉政建设和反腐败斗争，认真执行《廉政准则》和领导干部任期经济责任审计、一岗双责等规定，努力做到干部清正、政府清廉、政治清明。弘扬勤俭节约，反对奢侈浪费，以低廉的行政成本实现高效的公共服务，严格控制楼堂馆所建设，严格控制“三公”经费支出，严格规范财政和投资预算约束。凡财政性投融资项目大量超预算的，不允许擅自追加资金;凡行政事业单位修建办公用房，必须统筹规划建设，以节约土地资源和行政运行成本。强化权力运行监督，加大对重点领域、关键环节、重要岗位的监督力度，确保行政权力运行到哪里，监督就延伸到哪里。全面推行阳光政务，健全完善统一规范的公共资源交易市场，切实规范政府投资管理和工程项目招投标制度，坚决防止和查处工程建设领域的黑幕行为。推进权力运行的公开化和规范化，扩大群众的知情权和监督权，提高决策的透明度和公众的参与度，让政府在监督中履职，让权力在阳光下运行。

　　各位代表!全面建成小康社会，建设美丽岳阳、幸福岳阳，是时代赋予的光荣使命，是全市人民的共同追求，也是我们的神圣职责。让我们在中共岳阳市委的坚强领导下，紧紧依靠全市人民，凝聚力量，开拓创新，锐意进取，向全面建成小康社会的宏伟目标奋勇前进!
